# On-Demand Multi-Resolution Liquid Alloy Printing Based on Viscoelastic Flow Squeezing

**DOI:** 10.3390/polym10030330

**Published:** 2018-03-16

**Authors:** Kang Wu, Pan Zhang, Fen Li, Chuanfei Guo, Zhigang Wu

**Affiliations:** 1 State Key Laboratory of Digital Manufacturing Equipment and Technology, Huazhong University of Science and Technology, Wuhan 430074, China; wuk16@hust.edu.cn (K.W.); zhangpan0830@hust.edu.cn (P.Z.); fenli0751@gmail.com (F.L.); 2 Department of Materials Science & Engineering, Southern University of Science & Technology, Shenzhen 518055, China; guocf@sustc.edu.cn

**Keywords:** stretchable electronics, liquid alloy, printing, multi-resolution, squeezing effect

## Abstract

Recently, high-resolution patterning techniques of stretchable electronics advanced extensively. An important trend is to fabricate complex circuits with varied sizes in a small area, which is a technical challenge to current conductive ink printing technologies. Here, we introduce a new strategy for multi-resolution liquid alloy printing, which can tune the resolution of printed liquid alloy trace in real time with the squeezing effect of compound viscoelastic flow. A newly developed coaxial nozzle with the inner nozzle extension (CNINE) is used to wrap and squeeze liquid alloy steadily and effectively. By controlling the working parameters and compound flow properties, liquid alloy patterns with different widths are obtained continuously. This work offers a new way to rapidly manufacture complex stretchable electronics patterning in multi-resolution.

## 1. Introduction

Owing to their unique properties such as great compliance to human bodies, stretchable electronics have attracted intensive attention in recent years [1]. Various applications such as stretchable antennas [2], soft sensors [3,4], ultra-stretchable nanogenerator [5], and flexible microelectrodes [6] are demonstrated because of their elastic mechanical responses [7]. The materials and the processing technologies play important roles in the performance of the stretchability and reliability of stretchable electronics [8,9]. Typically, room temperature liquid alloy, one kind of intrinsically stretchable conductors, has been widely used due to their high conductivity [10], high stretchability, facile fabrication and integration [11]. Many processing technologies for liquid-alloy-based stretchable electronics such as microchannel injection [12,13], mask deposition [14,15,16] and micro-contact printing [17], are developed for high resolution applications. However, many processes during fabricating, such as photolithography, are not as convenient or cost-effective.

Without using of masks or stencils, the fabrication of stretchable electronics could be greatly simplified, such as by using inkjet printing [18,19], all-printing [20] and direct writing [21]. Among them, direct writing shows attractive features, such as convenience and customized prototyping capability [22]. Zheng et al. designed a roller-ball pen for rapid liquid alloy printing [23,24]. Boley et al. studied the characterization of directly printed liquid alloy traces and fabricated a strain gauge [25]. These works increase the efficiency for rapid manufacture. Nowadays, complex patterning is demanded in stretchable electronics to integrate more functional features into a small area. Thus, it is important to achieve high resolution printing and rapid manufacture simultaneously. Yan et al. fabricated a strain sensor via printing conductor and elastomer simultaneously using a coaxial nozzle where two outlets are coplanar [26]. Coaxial flow printing is a simple way that cannot only print multi-materials at the same time, but also control the inner flow by flow focusing [27,28]. Yuk et al. tried to break the limitation of existing Direct Ink Writing (DIW) 3D printing by harnessing deformation, instability and fracture of viscoelastic inks [29]. Therefore, it is necessary to investigate the behaviors of liquid alloy and elastomer flow in the coaxial flow field for resolution control during the printing.

**Figure 1:**
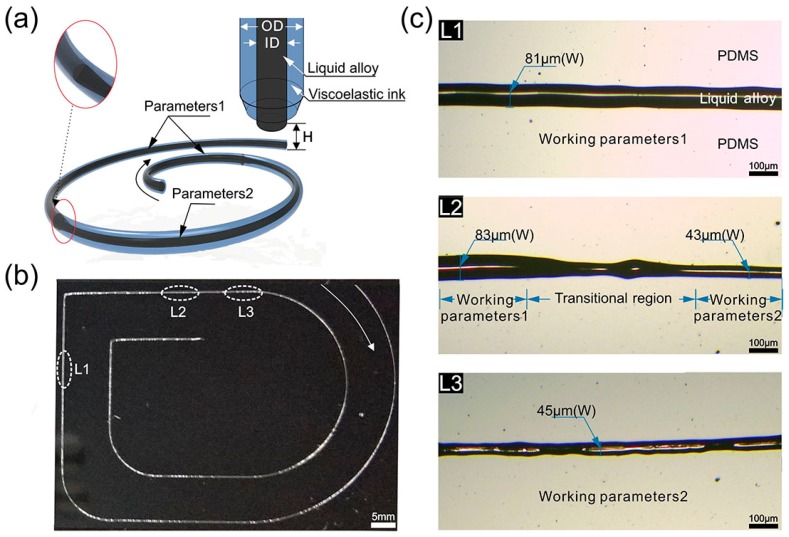
(**a**) Schematic illustration of liquid ally cable with different diameters during printing process; (**b**) photograph of printed cable with different diameters of liquid alloy; (**c**) optical images of different segments of the cable shown in Figure 1b: the diameters of liquid alloy in L1 and L3 are about 81 μm and 45 μm, respectively. L2 is the transitional region between L1 and L3. Working parameters 1: The moving speed of the stage (V_P_) is 25 mm/s, the flowrate of liquid alloy and PDMS are 8 µL/min (Q_ID_) and 80 µL/min (Q_OD_), respectively. Working parameters 2: The moving speed of the stage is 50 mm/s, the flowrate of liquid alloy and PDMS are 4 µL/min and 80 µL/min, respectively. The distance between the outlet of coaxial nozzle and substrate (H) is about 10 µm.

Here, we present a new strategy for tuning the printed liquid-alloy trace resolution in real time with compound viscoelastic flow squeezing, which can print a multi-resolution liquid alloy pattern on demand. As shown in Figure 1a, a liquid alloy trace with different sizes can be printed by using a newly designed coaxial nozzle, where the inner and outer channel flows were liquid alloy and viscoelastic flow, respectively. By using the squeezing effect of compound viscoelastic flow, a pattern with different widths of a liquid alloy cable was printed continuously on demand, as shown in Figure 1b,c. Moreover, the diameter of the liquid alloy cable can be tuned from 81 μm to nearly half as designed. Furthermore, the principle of compound viscoelastic flow squeezing on liquid alloy and corresponding working parameters that influence the printed width were studied. Finally, two flexibly selective heating devices were demonstrated using the newly developed printing technique.

## 2. Materials and Methods

### 2.1. Materials

Room temperature liquid alloy galinstan, GaInSn (68.5 wt% gallium, 21.5 wt% indium, 10 wt% tin, with viscosity of 2.4 × 10^−3^ Pa∙s, from Geratherm Medical AG, Geschwenda, Germany) was used as the inner flow. It was used without any pre-processing and post-processing [30,31].

A silicone (polydimethylsiloxane, PDMS) kit, Elastosil RT601 A and B (mixed at a weight ratio of 9:1, Wacker Chemie, Munchen, Germany) was used as outer channel flow. The diluted PDMS was prepared by adding 3 wt% thinner (Silicone Thinner, Smooth-On, Macungie, PA, USA) to the mixed PDMS. To tune the surface tension, a 0.33 wt% surfactant Silwet L-77 (GE, Boston, MA, USA) was added to the PDMS mixture. Afterwards, the whole mixtures were put in vacuum for 10 min to eliminate bubbles. Ecoflex 0030 (Smooth-On, Macungie, PA, USA), another kind of silicone kit (mixed at a weight ratio of 1:1), was used to improve the stretchability of printed devices.

The viscosities of pure PDMS, diluted PDMS and surfactant added PDMS are 4.73 Pa∙s, 4.39 Pa∙s and 13.2 Pa∙s, respectively, which are measured by a rheometer (DHR-1, TA, New Castle, DE, USA) after the silicones were prepared for 10 min. All measurements were conducted at 20 °C with preliminary equilibration time of 1 min.

### 2.2. Device Fabrication and Assembly

**Figure 2:**
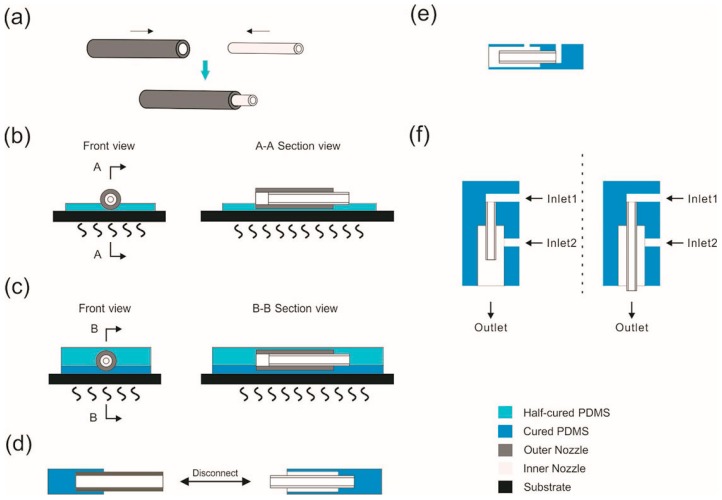
Fabrication of a coaxial nozzle. (**a**) Two steel tubes with different diameters were nested; (**b**) the nested tubes were placed horizontally in surface cured PDMS which was totally cured afterwards; (**c**) the nested tubes covered by liquid PDMS were heated to cure PDMS and then the tubes were encapsulated; (**d**) the outer tube was removed from the cured structure and the left space was treated as the outer channel, while the inner channel was set up by the unmoved inner tube; (**e**) the entrances of the inner flow and outer flow were punched out by a specific tube; (**f**) cross sections of the coaxial nozzle with the inner nozzle clipped (CNINC) and CNINE.

Coaxial nozzle fabrication: a simplified fabrication process of coaxial nozzle was shown in Figure 2. First, two steel tubes with matched diameter were nested together. Afterwards, liquid PDMS was poured onto a petri dish for about 5 mm thick and then put into an oven (UF-55, Memmert, Germany) at 75 °C for 2 min to cure the surface part of the whole PDMS mixture. Next, the nested tubes were placed horizontally on the surface of PDMS immediately, and then the whole structure was covered with another layer of 5 mm thick of uncured PDMS. Following that, the petri dish was put into the oven at 75 °C for 5 min to cure the whole PDMS thoroughly. Afterwards, the outer tube was removed from the cured structure and the left space was used as an outer channel, while the inner steel tube served as an inner channel. Finally, the entrances of the inner flow and outer flow were punched by a specific needle, respectively. The length of outer channel could be adjusted as required.

Printing System: two identical syringe pumps (PUMP 11 ELITE Nanomite, Harvard Apparatus, Holliston, MA, USA) were used to prime liquids through coaxial nozzles. An XYZ platform (SM200DS, MUSASHI, Musashino, Tokyo, Japan) was employed to print circuits by controlling the relative position of nozzle and substrate. A camera (Y002, Supereyes, Shenzhen, China) was used to monitor the printing process, as shown in Figure S1. H could be controlled accurately via adjusting the height of platform. When studying the influence of other relevant parameters on liquid alloy trace width (W), H was remained constant (=10 µm).

Post-processing: For the fabrication process of devices, liquid alloy-in-PDMS wires and its wafer substrate were heated for 5 min at 75 °C to fully cure PDMS after printing.

### 2.3. Data Acquisition and Post-Processing

The width of liquid alloy cable, W, was taken as the characteristic parameter to analyze the quality of circuit. In the experiments, for each value of specific parameter, three cables were printed and each cable was captured at 15 different segments by a metallographic microscope (BA310MET-T, Motic, Xiamen, China), as shown in Figure S2. W was acquired by using a self-developed program based on Matlab (Version 2016b, MathWorks, Natick, MA, USA) to compute the mean value of the widths of 45 segments. The numerical simulations were conducted by COMSOL (COMSOL Multiphysics 5.2, Stockholm, Sweden).

An electromechanical testing machine (E1000, Instron, Boston, MA, USA) and a digital multimeter (34461A, KeySight Technologies, Santa Rosa, CA, USA) were used for cycling test. An infrared radiation (IR) thermal imaging camera (T420, FLIR Systems, Inc., South San Francisco, CA, USA) was used for IR thermograph capture.

## 3. Results and Discussion

### 3.1. The Principle of Compound Viscoelastic Flow Squeezing on Liquid Alloy

**Figure 3:**
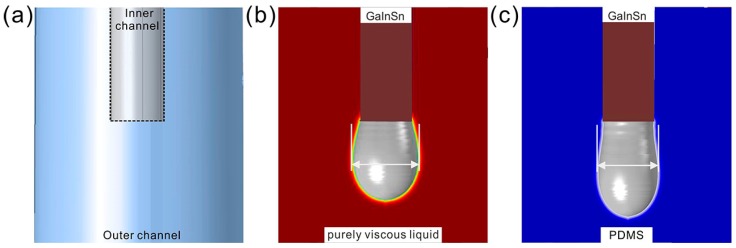
Simulated behavior of liquid alloy in the coaxial nozzle. (**a**) The model of flow field; (**b**) simulated behavior of liquid alloy with a purely viscous outer flow with the same viscosity as the PDMS; (**c**) simulated behavior of liquid alloy with PDMS squeezing. The flow rates of GaInSn and outer channel flow are 16 µL/min and 80 µL/min, respectively. The diameters of the inner channel and outer channel are 210 µm and 800 µm, respectively.

Figure 3 shows the simulated results of the behavior of liquid alloy in a coaxial nozzle with purely viscous liquid and PDMS. The liquid alloy goes in the inner nozzle and the other flow goes in the outer channel. It indicates that with purely viscous outer flow, liquid alloy swells as a sphericity with a bigger diameter than that of the inner channel (ID) when it is extruded out of the channel due to its high surface energy. However, when PDMS is introduced as viscoelastic outer flow at the same flowrate, the extruded liquid alloy from the inner channel is like an inverted conic and its diameter is a little bit smaller than ID. Extruded swelling is a common phenomenon in viscoelastic ink printing, it compressed into a die followed by a partial recovery to the former shape and volume of the fluid after exit [29,32]. Through introducing viscoelastic flow as outer flow, the inner flow liquid alloy is squeezed. A similar phenomenon was observed when introducing Ecoflex 0030.

**Figure 4:**
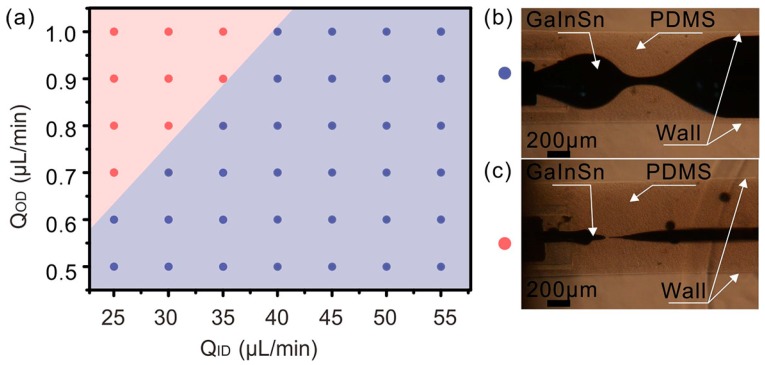
The flow behavior of liquid alloy in CNINC. (**a**) The distribution of instability states in CNINC; (**b**) if Q_ID_/Q_OD_ ≥ 40 approximately, the GaInSn flow keeps continuous but not uniform and the flowrate of outer flow PDMS is relatively little so that it cannot wrap the inner flow GaInSn completely, which is marked with blue dot; (**c**) the GaInSn flow is pinched off by PDMS flow when Q_ID_/Q_OD_ < 40, which is marked with red dot. Upper right: Q_ID_ = 45 µL/min, Q_OD_ = 1 µL/min. Lower right: Q_ID_ = 30 µL/min, Q_OD_ = 1 µL/min.

In order to print high-resolution liquid alloy trace, the squeezing effect of viscoelastic flow is introduced. Two kinds of coaxial nozzles are fabricated: CNINC and CNINE, as shown in Figure 2f. The two coaxial nozzles were fabricated with the same inner and outer channel size except the relative position of inner outlet and outer outlet. The behavior of liquid alloy in CNINC is shown in Figure 4. When a co-axial flow field was constructed by CNINC, there were two typical kinds of instabilities: the liquid alloy shrunk to droplets at a relative large flow rate and was pinched off by the PDMS at a relative small flow rate. The simulated droplet generation process implies the developing of flow instability, Figure S3. Due to the high surface energy of liquid alloy, it is difficult to keep inner flow continuous when the two flows met because of the Rayleigh-Plateau instability [33,34]. Moreover, if the Q_ID_/Q_OD_ is too large, it was hard for outer PDMS flow to wrap inner GaInSn flow completely. Therefore, the flow stability limited the application of CNINC in printing. Moreover, the squeezing effect on liquid alloy not only happened in nozzle but also existed along the printed trace out of the nozzle. For CNINE, the liquid alloy was squeezed at the same time with trace printing. Stable coaxial cables can be printed continuously when using CNINE even when the ratio of Q_ID_ to Q_OD_ is less than 0.1 (see Figure S2 for photograph of printed coaxial cables). Therefore, CNINE is chosen for further printing due to the operation convenience. In this paper, the inner channel of CNINE was extended out 500 µm compared to the outer channel.

**Figure 5:**
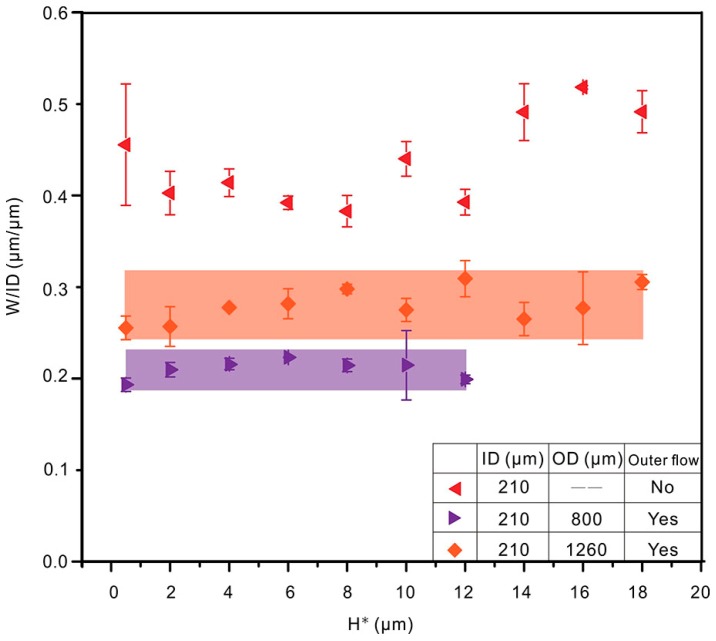
Trace widths normalized by ID as a function of H* and 95% confidence interval of widths printed by CNINE. A uniaxial nozzle and two CNINE with different outer channel diameters were used for the study (Q_ID_ = 8 µL/min, V_P_ = 40 mm/s). H* is the relative distance between the outlet of coaxial nozzle and its baseline (H* = H − 5 μm).

Figure 5 shows the ratio of W to inner diameter (W/ID) for traces printed onto a silicon wafer using three nozzles (a uniaxial nozzle and two CNINE with different outer channel diameters) with different values of H. W/ID is more than 0.35 (red) when using the uniaxial nozzle, and it reduces to less than 0.3 (orange) via introducing the outer flow, which agrees with the simulation results. Moreover, at the same ID and outer channel fluid speed (V_OD_), W/ID decreases to around 0.2 (purple) when using the coaxial nozzle with smaller outer channel diameter (OD). It implies that smaller W could be achieved via smaller ODs.

Meanwhile, the influence of H on W is studied. Without outer flows, the width of GaInSn trace is easily affected by the value of H, while the fluctuation of W reduces to a small range when an outer channel flow is introduced. While with a CNINE with smaller OD, the variation of W becomes smaller. All these results indicate that CNINE can not only be used to obtain smaller W, but it also reduces the influence of H. Further, variable parameters can be controlled to print different diameters of liquid alloy in coaxial cable.

### 3.2. The Influence of Relevant Parameters on Trace Width

**Figure 6:**
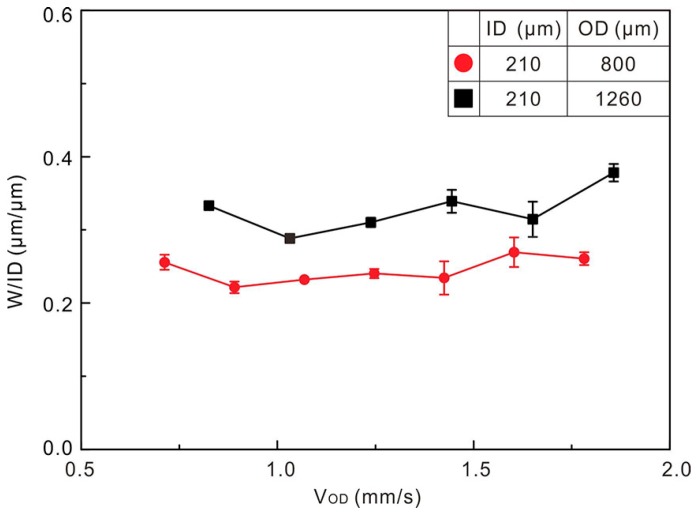
Trace widths normalized by ID as a function of outer channel flow speed. Two CNINE with different outer channel diameters were used (Q_ID_ = 8 µL/min, V_P_ = 40 mm/s).

The parameters including V_OD_, platform moving speed (V_P_), Q_ID_ and characteristics of outer flow are discussed to discover the influence of coaxial direct writing parameters on W. Normalized widths of liquid alloy traces are shown in Figure 6 with V_OD_ varying from around 0.7 to 1.9 mm/s. V_OD_ is defined as,

(1) $$V_{\mathrm{OD}}=\frac{{4Q}_{\mathrm{OD}}}{\pi ({\mathrm{OD}}^{2}-{\mathrm{ID}}^{2})}$$

For a given OD, the width fluctuates in a small range with the increasing flow speed. When W/ID increases from ≈0.25 to ≈0.33, the OD goes up from 800 μm to 1260 μm. This phenomenon implies that OD is a key factor on the traces width and that smaller OD provides stronger squeezing effect on the inner liquid alloy. Hence, narrower liquid alloy traces can be printed by reducing the outer channel size.

**Figure 7:**
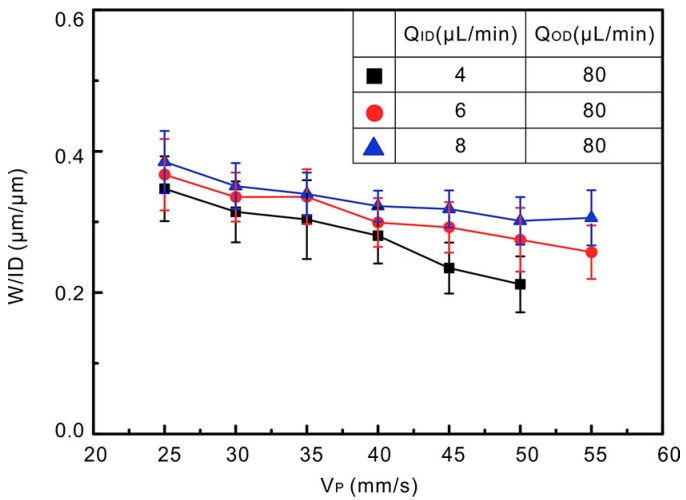
Trace widths normalized by ID as a function of the platform speed. Three different ratios of Q_ID_/Q_OD_ were used for study (ID = 210 µm, OD = 800 µm). There is no continuously trace when the moving speed is over 50 for small liquid alloy flow rate (black squares).

Figure 7 shows the effect induced by the movement speed of XYZ platform. For a given Q_ID_, W/ID decreases as the platform speeds up. As observed, at the same V_P_, following the decreasing of Q_ID_, W/ID decreases. This implies that both the platform moving speed and inner liquid flowrate play important roles on the width of liquid alloy trace.

**Figure 8:**
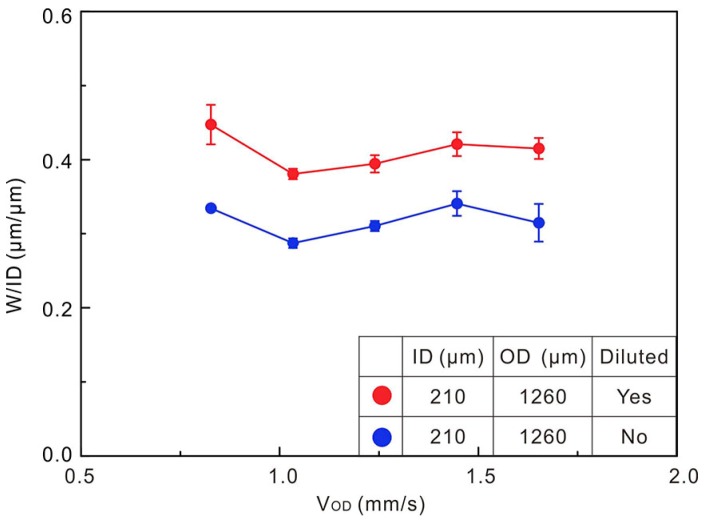
Trace widths normalized by ID as a function of outer channel flow speed with and without diluted outer flow (Q_ID_ = 8 µL/min, V_P_ = 40 mm/s).

The silicone thinner was used to increase the fluidity of PDMS. Figure 8 shows the influences on W of the pure PDMS and diluted PDMS. The trace printed with diluted PDMS as outer channel flow is wider (W/ID > 0.35) than that using the pure PDMS (W/ID < 0.35). It indicates that the outer flow viscosity has a significant influence on the trace width and the outer flow with lower viscosity weakens the squeezing effect on inner flow.

**Figure 9:**
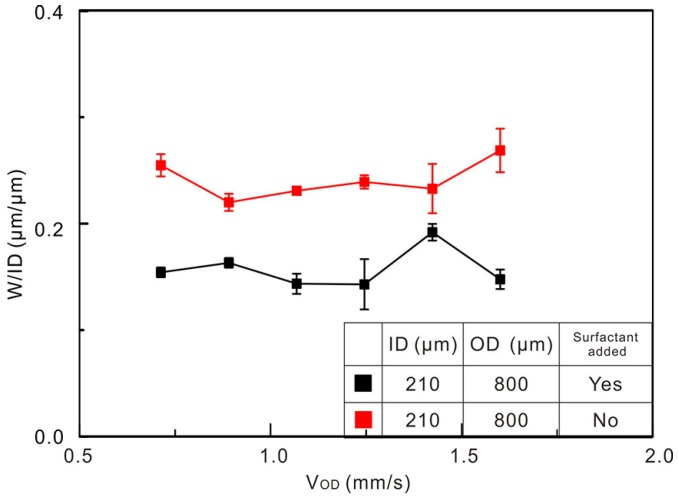
Trace widths normalized by ID as a function of outer channel flow speed with and without surfactant added outer flow (Q_ID_ = 8 µL/min, V_P_ = 40 mm/s).

Furthermore, we used a silicone-based surfactant to reduce the liquid-alloy/PDMS interfacial energy gradient. Figure 9 shows the comparison between the pure PDMS and the surfactant added PDMS. The result demonstrates that the trace printed with surfactant added PDMS is narrower (W/ID < 0.2) than that using a pure one (W/ID > 0.2).

### 3.3. Stretchable Strain Sensor by Coaxial Printing

**Figure 10:**
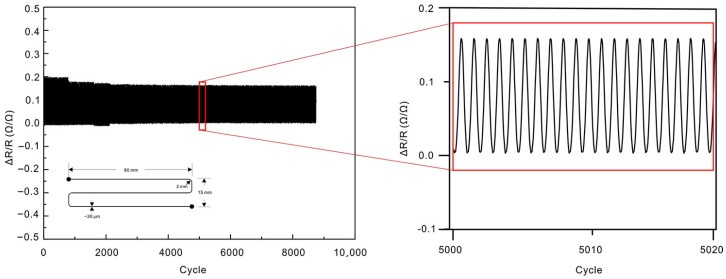
Results of resistance for encapsulated strain sensor in tensile tests. The strain sensor can be stretched more than 25% for nearly 9000 cycles. As the strain at X and Y direction was a bit different during the test, the liquid alloy reflowed within the PDMS channel due to its high fluidity, and a tiny width and height change was happened along the channel. Therefore, the resistance variation during the first 2000 cycles is a little higher before dynamic balance of liquid metal distributions achieved.

A stretchable strain sensor with an S-shaped cable was fabricated using the coaxial direct writing (see Figure S4 for fabrication process). The width of GaInSn in each wire is around 30 μm and the whole device is about 80 mm long and 15 mm wide. The strain sensor can be stretched up to nearly 100% without breakup and the stretchability can be easily improved by using Ecoflex 0030 instead of PDMS. The strain sensor can be elongated more than 25% for nearly 9000 cycles, as shown in Figure 10, which demonstrates high sensitivity as well as high reliability of electronics fabricated by this processing technique (see Figure S5 for photograph of cycling testing).

### 3.4. Flexibly Selective Heating Devices

**Figure 11:**
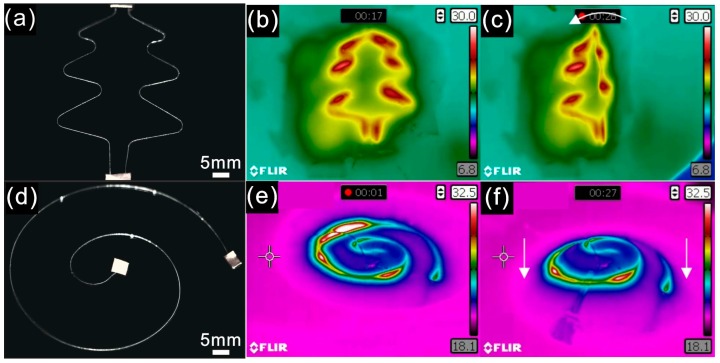
Printed structures with different diameters of liquid alloy: (**a**) printed tree structure heating device; (**b**) infrared thermography of tree structure heating device when applied voltage is 2 V; (**c**) infrared thermography of tree structure heating device after bending 90°; (**d**) helix structure heating device; (**e**) infrared thermography of helix structure heating device when the applied voltage is 2.5 V; (**f**) infrared thermography of helix structure heating device after stretching.

As shown in Figure 11a,d, two flexibly selective heating devices were fabricated, through tuning different parameters, where the width of liquid alloy can be adjusted in real time on demand within a single coaxial cable. Compared to the wider segment of liquid alloy, the narrower one generates more heat and the temperature is higher after applying voltage. Therefore, a flexible selective heating stages can be obtained in a single step processing. As shown in Figure 11a,d, clear temperature differences in a small area were observed in the IR thermographs. Figure 11c shows the tree structure heating device after bending 90° and Figure 11f shows the helix structure heating device after applying load downwards at its edge. The colors of the structures are stable during the deformation, which indicates no obvious change in width, as shown in Video S1 and Video S2. This implies that a flexible device with precise heating variation in a small area even less than 10 mm × 10 mm can be achieved using our technique.

## 4. Conclusions

In summary, we present a new strategy for multi-resolution liquid alloy printing, which can tune the printed liquid-alloy trace resolution on demand. We studied the principle of compound viscoelastic flow squeezing on liquid alloy and then developed a new coaxial nozzle with the inner nozzle extension (CNINE) to wrap and squeeze liquid alloy steadily and effectively. A fine liquid alloy trace can be obtained by using smaller outer channel with surfactant introduced viscoelastic flow. With proper printing parameters such as flow rate and platform moving speed, a multi-resolution pattern can be achieved continuously. This technology provides a new approach for rapid and flexible manufacture of complex stretchable electronics or soft systems.

## Supplementary Materials

The following are available online at http://www.mdpi.com/2073-4360/10/3/330/s1, Figure S1: Photograph of printing system; Figure S2: Schematic illustration of data acquisition and photograph of printed coaxial cables; Figure S3: Simulation of the flow instability; Figure S4: Fabrication process of stretchable electronic device; Figure S5: Photograph of stretchability testing system; Video S1: IR thermograph video of the deformation of the tree structure; Video S2: IR thermograph video of the deformation of the helix structure.
