# Supplementary material for: On-Demand Multi-Resolution Liquid Alloy Printing Based on Viscoelastic Flow Squeezing
Source: Polymers (Basel). 2018 Mar 16;10(3):330. doi: 10.3390/polym10030330 (PMC6414868; doi:10.3390/polym10030330)
Supplement: Supplementary file 1 [file polymers-10-00330-s001.zip › SI/SI-1.docx]

Supplementary Information

On-demand Multi-Resolution Liquid Alloy Printing based on Viscoelastic Flow Squeezing

Kang Wu ^1,+^ , Pan Zhang ^1,+^ , Fen Li^1^, Chuanfei Guo^2^ and Zhigang Wu^1,^*

^1^ State Key Laboratory of Digital Manufacturing Equipment and Technology, Huazhong University of Science and Technology, Wuhan 430074, China; wuk16@hust.edu.cn (K.W.); zhangpan0830@hust.edu.cn (P.Z.); fenli0751@gmail.com (F.L.)

^2^ Department of Materials Science & Engineering, Southern University of Science & Technology, Shenzhen, Guangdong 518055, China; [guocf@sustc.edu.cn (C](mailto:guocf@sustc.edu.cn%20(C).G.)

+ Kang Wu and Pan Zhang contributed equally to this work.

***** Correspondence: zgwu@hust.edu.cn; Tel.: +86-27-8754-4054

**Figure S1.** Photograph of the printing system including XYZ platform, two syringe pumps, an optical imaging camera connected to PC and CNINE.

**Figure S2.** Schematic illustration of data acquisition and photograph of printed coaxial cables. For each value of a specific parameter, three cables were printed and each cable was captured at 15 adjoining segments. Each segment is 2 mm long and 1.5 mm wide. W was acquired via computing the mean value of the widths of 45 segments. All the data in this work were captured in the stable segments to avoid the start/end instability.

**Figure S3.** Simulation of the flow instability. Liquid alloy flow necked in the PDMS induced by the Rayleigh-Plateau instability. A liquid alloy droplet generated due to the flow instability developing with time (Q_ID_= 35 µL/min, Q_OD_= 1 µL/min).

**Figure S4.** Fabrication process of the stretchable sensor. Firstly, coaxial cable was printed on a PDMS substrate; then, it was heated to cure the PDMS; finally, metal wires were used to connect the two ends of the cable.

**Figure S5.** Photograph of the cycling testing system including an electromechanical testing instrument and a digital multi-meter.
